# Acute/subacute paracoccidioidomycosis associated with drug-resistant tuberculosis in a person living with HIV/AIDS

**DOI:** 10.1590/S1678-9946202668004

**Published:** 2026-01-30

**Authors:** Lívia Novaes Teixeira, Nicolas de Albuquerque Weidebach, Ana Angélica Bulcão Portela Lindoso, Cesar Cilento Ponce, José Angelo Lauletta Lindoso

**Affiliations:** 1 Instituto de Infectologia Emilio Ribas São Paulo Brazil Instituto de Infectologia Emilio Ribas, São Paulo, São Paulo, Brazil; 2 Universidade Metropolitana de Santos Disciplina de Patologia São Paulo Brazil Universidade Metropolitana de Santos, Disciplina de Patologia, Santos, São Paulo, Brazil; 3 Universidade de São Paulo Instituto de Medicina Tropical de São Paulo Faculdade de Medicina São Paulo Brazil Universidade de São Paulo, Faculdade de Medicina, Instituto de Medicina Tropical de São Paulo, Laboratório de Protozoologia, São Paulo, São Paulo, Brazil

**Keywords:** Paracoccidioidomycosis, HIV, Immunosuppression, Tuberculosis drug-resistant

## Abstract

Paracoccidioidomycosis (PCM) is a neglected tropical disease classified as acute/subacute and chronic. In people living with HIV/AIDS (PLWHA), coinfection can lead to severe clinical manifestations. We report the case of a 30-year-old immunosuppressed male presenting fever, weight loss, polymorphic skin lesions, diffuse lymphadenopathy, hepatosplenomegaly, and joint effusion. Histopathological analysis revealed fungal structures compatible with *Paracoccidioides* spp., and serology was positive at a titer of 1:16. Despite initial Amphotericin B and antiretroviral therapy, the patient developed a productive cough and persistent systemic symptoms. Initial sputum tests were negative for *Mycobacterium tuberculosis*, but subsequent bronchoalveolar lavage detected rifampin-resistant tuberculosis (TB). The remarkable overlap of clinical and radiological features of TB and PCM can significantly delay diagnosis, highlighting the need for high clinical suspicion and prompt investigation with bronchoalveolar lavage (BAL) testing. After one-month outpatient follow-up, the patient showed significant cutaneous improvement, undetectable HIV viral load, and a marked increase in CD4+ T-cell count. This report highlights the importance of recognizing the acute/subacute form of PCM as an AIDS-defining illness in endemic areas, enabling early treatment and improved outcomes.

## INTRODUCTION

Paracoccidioidomycosis (PCM) is a systemic mycosis of significant public health importance, endemic in Latin America, with Brazil reporting the majority of cases^1^. Despite its epidemiological relevance, true disease burden remains underestimated, as PCM is not included in the Brazilian list of notifiable diseases^1,2^. Notably, PCM is one of the leading causes of death among chronic infectious diseases in Brazil^2,3^.

PCM presents in two distinct forms: acute/subacute and chronic. The first typically involves the mononuclear phagocyte system, manifesting with generalized lymphadenopathy, hepatosplenomegaly, fever, and frequently cutaneous lesions due to hematogenous dissemination^4^. Conversely, the chronic form usually emerges years after primary infection, predominantly affecting the lungs but potentially involving the larynx, upper respiratory tract, and central nervous system^4^.

The clinical spectrum of PCM is closely related to the host's immune response^5^. A predominant T-helper 2 (Th2) immune profile, associated with humoral immunity, facilitates fungal proliferation and is typically associated with the acute/subacute form, predominantly affecting children and young adults^4,5^. In contrast, a T-helper 1 (Th1)-mediated cellular response promotes fungal containment and is characteristic of the chronic form^4,5^. In immunosuppressed patients, particularly those with impaired cell-mediated immunity, latent *Paracoccidioides* spp. infection may reactivate, leading to clinical manifestations that resemble the acute/subacute presentation^3,5-9^.

The first reported association between PCM and HIV/AIDS occurred in 1989^10^. By 2018, only 136 cases had been reported in PLWHA worldwide^11^. Profound depletion of CD4+ T-cells in this population often results in atypical and disseminated disease, leading to delayed diagnosis and poor therapeutic outcomes^3,6^.

The association between PCM and tuberculosis (TB) is well recognized, as both conditions share overlapping clinical and radiological features that can hinder the diagnosis^4,12-14^. The prevalence of TB among PCM patients ranges from 2% to 20%^4,12^. However, reports of rifampin-resistant TB co-infection in PLWHA with PCM remain rare^14^.

We describe a case of acute/subacute PCM in a PLWHA, presenting with reticuloendothelial and cutaneous involvement, concomitant with rifampin-resistant pulmonary TB.

### Ethics

This study was approved by the Research Ethics Committee of the Institute of Infectology Emilio Ribas (report Nº 7.370.274).

## CASE REPORT

A 30-year-old white male security guard from Sao Paulo's metropolitan region, Sao Paulo State, Brazil, presented to the emergency department reporting the onset of multiple facial papular lesions approximately 45 days before admission, with subsequent dissemination to the chest, back, abdomen, and limbs. Additional symptoms included fever, night sweats, chills, dry cough, anorexia, and unintentional weight loss of 10 kg. He had no odynophagia, dysphagia, diarrhea, or abdominal pain. Approximately 10 days after symptom onset, he developed lower limb weakness and gait impairment due to bilateral knee arthralgia, more pronounced on the left side. The patient was born in the municipality of Osasco and denied having occupational or recreational soil exposure, agricultural activities, or rural residence. He lived with healthy cats and no contact with other animals. There was no history of travel, smoking, or alcohol use. HIV infection had been diagnosed five years earlier; however, the patient had discontinued antiretroviral therapy (ART) one year before admission.

**Figure 1:**
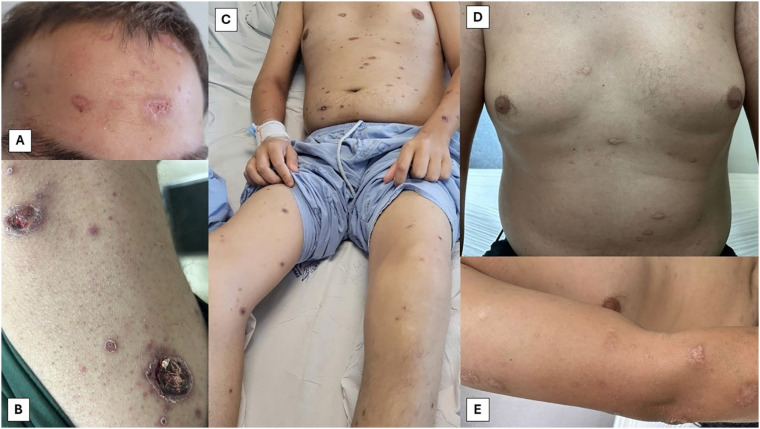
Skin lesions: (A) Shallow ulcers in the frontal region; (B) Multiple papular lesions surrounding two ulcerative lesions with crust formation and central necrosis on the lateral face of the right thigh; (C) Disseminated small ulcerative lesions on the torso, upper and lower extremities. Significant edema in the left knee; (D and E) Cicatricial lesions on the torso and upper left arm, one month after hospital discharge and follow up.

He was hemodynamically stable and exhibited disseminated, pleomorphic cutaneous lesions, including ulcerated-crusted plaques, macules, and papules with central necrosis (Figures 1A, 1B and 1C). Mobile, fibroelastic, bilateral and slightly tender lymphadenopathy was palpable in the submandibular, axillary, and inguinal regions. The liver was palpable 4 cm below the right costal margin and the spleen, 3 cm below the left costal margin. The left knee was swollen, warm, and painful upon flexion.

**Figure 2:**
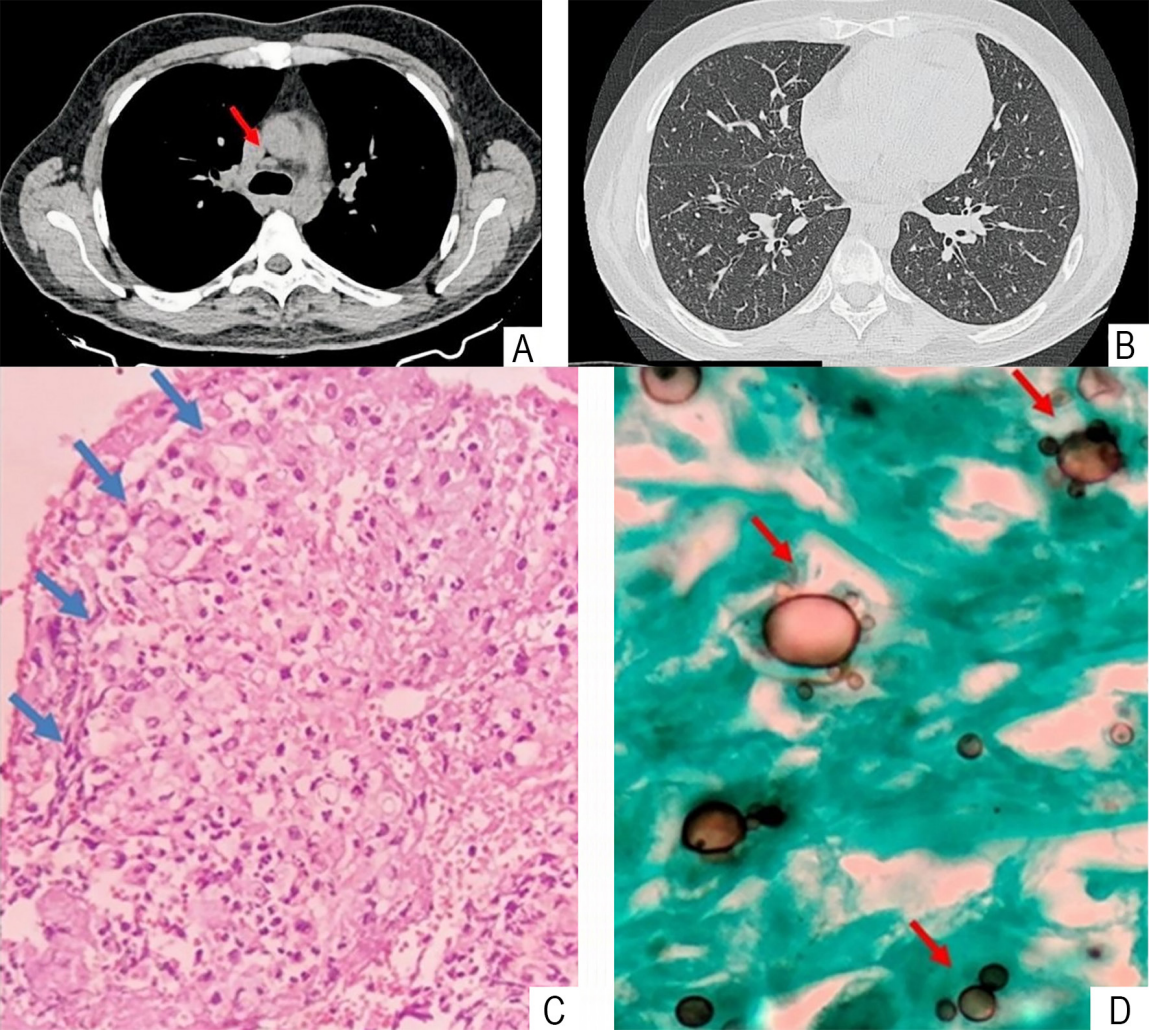
Computerized tomography chest (CT) scan: (A) Prominent paratracheal enlarged lymph node in the mediastinal window (red arrow); (B) Diffuse reticulo-micronodular opacities throughout the lung parenchyma associated with diffuse bronchial thickening. Skin lesion biopsy histopathological analysis: (C) Chronic granulomatous dermal inflammation with Hematoxylin and Eosin stain (blue arrows) (200x); (D) Fungal yeasts with great variability in size and multiple exosporation (red arrows), compatible with *Paracoccidioides spp*. (Grocott-Gomori methenamine silver stain = Grocott-Gomori methenamine silver, 1000x).

Initial laboratory findings showed a CD4+ T-cell count of 4 cells/mm^3^ and *HIV-1 viral load* of 271,000 copies/mL. Hemoglobin was 9.8 g/dL, leukocyte count of 6,900/mm^3^, platelet count of 458,000/mm^3^, AST 98 U/L, ALT 48 U/L, and C-reactive protein of 249 mg/L. Sputum smear microscopy and molecular testing for *Mycobacterium tuberculosis* were negative. Blood tests for cryptococcal antigen, syphilis, hepatitis A/B/C, and histoplasmosis were also negative. Cytomegalovirus (CMV) serology showed negative IgM, positive IgG, and a quantitative plasma DNA load of 297 IU/mL. Chest computed tomography (CT) revealed right paratracheal lymphadenopathy, bilateral axillary chains, micronodular opacities, and bronchial thickening (Figure 2A and 2B). CT of the left lower limb demonstrated moderate joint effusion and anterior soft tissue edema, with preserved articular spaces.

Histopathological examination of a skin biopsy revealed chronic granulomatous dermatitis. Hematoxylin-eosin staining showed granulomatous inflammation, while Grocott-Gomori methenamine silver (GMS) staining showed multiple budding yeast forms compatible with *Paracoccidioides* spp. (Figures 2C and 2D). Double agar gel immunodiffusion serology for PCM was positive at a titer of 1:16.

The patient was initially treated with intravenous amphotericin B deoxycholate (1 mg/kg/day for 13 days), followed by amphotericin B lipid complex (5 mg/kg/day) for a total of 5 weeks, reaching a cumulative dose of 100 mg/kg. ART was initiated on the 8th day of hospitalization, comprising tenofovir, lamivudine, and dolutegravir. Despite both therapies, the patient remained febrile and developed a productive cough, accompanied by new pulmonary auscultation findings. A chest CT scan revealed diffuse pulmonary micronodules. Repeated sputum smears and molecular assays for *M. tuberculosis* remained negative. Consequently, bronchoscopy was performed, and a molecular tuberculosis test (GeneXpert MTB/RIF, Cepheid, USA) on BAL fluid detected low levels of *M. tuberculosis* with positive rifampin resistance gene. The BAL Ziehl-Neelsen staining and mycobacterial culture were negative. A multidrug-resistant tuberculosis regimen was initiated with bedaquiline, levofloxacin, linezolid, and terizidone. The patient exhibited clinical improvement, with resolution of fever and respiratory symptoms.

**Figure 3:**
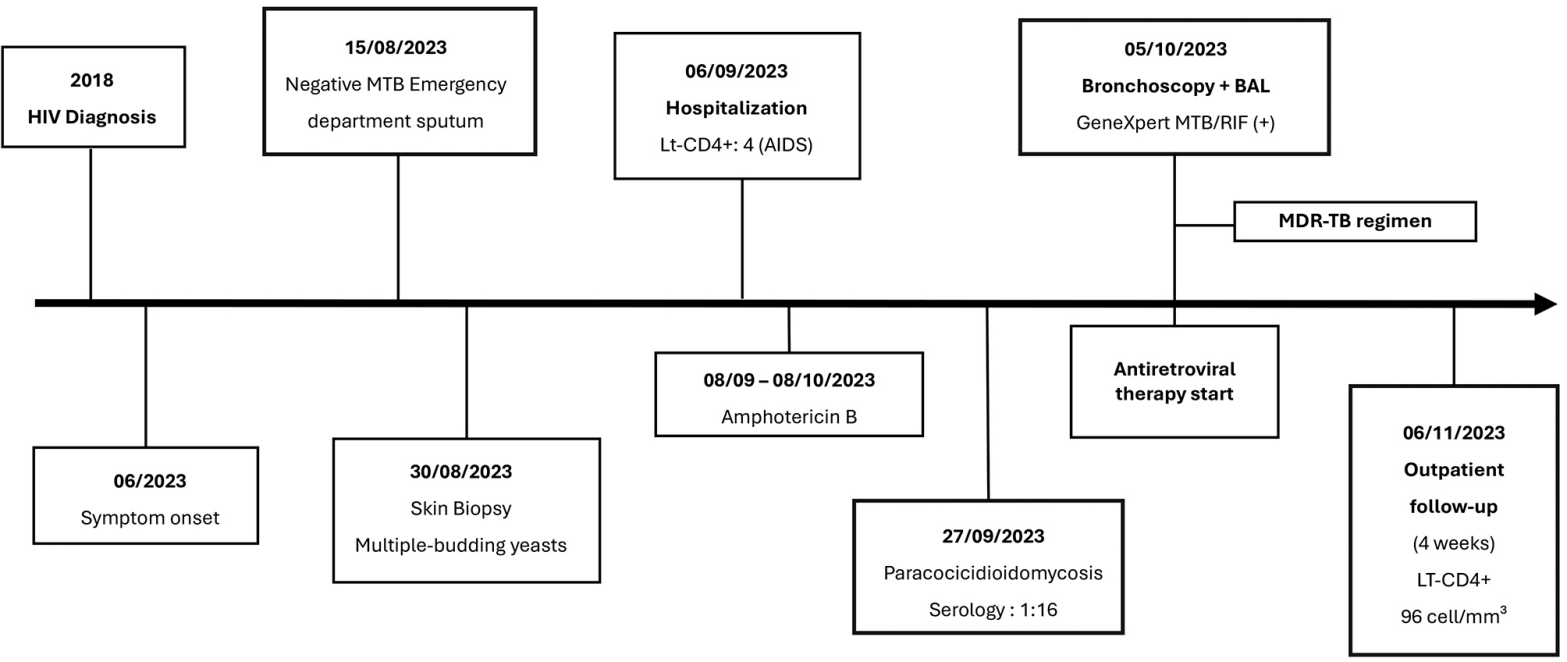
Timeline of the patient's clinical course and therapeutic interventions, illustrating symptom onset, diagnostic milestones, and key treatment decisions.

After 42 days of hospitalization, he was discharged afebrile and asymptomatic. Oral trimethoprim-sulfamethoxazole (800/160 mg twice daily) was prescribed for secondary prophylaxis of PCM and primary prophylaxis against toxoplasmosis and pneumocystosis. After one-month outpatient follow-up, the patient demonstrated complete resolution of cutaneous lesions (Figure 1D and 1E), an undetectable HIV viral load, and a CD4+ T-cell count of 96 cells/mm³. A timeline shows the clinical outcome in detail (Figure 3).

## DISCUSSION

In this report we describe a paracoccidioidomycosis case with acute/subacute clinical features, associated with rifampin-resistant pulmonary tuberculosis, in a PLWHA with severe immunodeficiency, characterized by a low CD4+ T lymphocyte count. This scenario highlights the importance of the host's cellular immune response and shows the natural course of this tropical fungal disease^10^. In immunocompetent individuals, a predominant Th2 response favors a primary acute/subacute form of PCM. However, in the context of profound immunosuppression, the pathogen behaves as an opportunistic infection^9^. We characterize it as a reactivation of latent *Paracoccidioides* spp., possibly acquired during childhood or adolescence, caused by uncontrolled HIV infection, inadequate treatment, and CD4+ T-cell depletion, favoring yeast proliferation and hematogenous dissemination^15^. In HIV/PCM coinfected patients, a histopathological similarity to the acute form of PCM can also be observed. Cellular immunosuppression impairs granuloma formation—the main host defense against *Paracoccidioides* spp.—thereby facilitating fungal proliferation, immune evasion, and marked clinical manifestations^6^. Accordingly, our patient exhibited reticuloendothelial involvement, polymorphic skin lesions, and joint symptoms, mimicking the acute/subacute form. In a cohort of 31 PCM/HIV cases, lymphatic involvement (54.8%), polymorphic skin lesions, and severe disease progression were common. Fever (67.8%) and weight loss (64.5%) were frequent, while mucosal lesions were less common^3^.

Individuals with compromised cellular immune responses are more susceptible to tuberculosis and other diseases caused by fungi, protozoa, and other mycobacteria^12^, as in the case reported here. The overlapping clinical and radiological findings of TB and PCM may hinder timely and accurate diagnosis, especially in settings with limited resources^12^. One case report from Peru has described PCM coinfected with multidrug-resistant TB, in which the patient had poor outcomes due to other concomitant infections^14^. To our knowledge, this is the first reported case of PCM coinfected with rifampicin-resistant TB with a favorable outcome. Healthcare professionals must be aware of the risk of this co-occurrence in PLWHA in endemic areas, as inadequate treatment increases the risk of developing pulmonary sequelae, leading to a reduction in quality of life^12^. Paniago *et al.*^13^ observed coinfection in 7 out of 12 cases involving PLWHA^13^. As with our patient, a thorough investigation should be performed, especially when symptoms persist during antifungal treatment, including both sputum and BAL tests. During follow-up of the reported case, respiratory and systemic symptoms persisted, prompting a detailed investigation for pulmonary tuberculosis with sputum samples and bronchoscopy with BAL testing, which detected rifampin-resistant *M. tuberculosis* DNA. The significant clinical improvement after starting treatment for tuberculosis supports the presence of both infections in this patient and highlights the unusual but concerning occurrence of drug-resistant TB in PCM-HIV scenarios.

The diagnosis of acute/subacute PCM is facilitated by the high pathogen load, which allows the detection of characteristic fungal structures in superficial lesions, either through direct examination or histopathological analysis of biopsies^4^. In this case, the identification of such structures was eased by acute presentation features in a patient with severe immunosuppression. In a retrospective study in Argentina, Messina *et al*.^16^ demonstrated that direct microscopic examination of skin, mucosal, or respiratory lesions has high sensitivity, especially in PLWHA, reflecting higher fungal burden^16^. Serological testing, however, has shown conflicting results. High sensitivity has been reported in one retrospective study^16^. On the other hand, lower seropositivity rates have been observed in PLWHA compared to non-HIV-infected individuals^7,17^. Despite its lower sensitivity, serological testing may still be useful for diagnosis^4,9^. In the present case, serology was positive, which may reflect the patient's humoral memory, with probable previous infection and reactivation in the context of cellular immunosuppression.

Studies have not shown a higher incidence of PCM in PLWHA, as with other invasive fungal diseases^3^. This dissociation, however, may be explained by the common use of *Paracoccidioides* spp. active drugs in this population, such as cotrimoxazole and azole derivatives^3^. Data on treatment of PCM in PLWHA remains scarce^18^. It has been observed that in PLWHA, the mortality rate for PCM is 35%, while in non-HIV-infected individuals, this rate is 7.9%^7^. It is known that amphotericin B should probably be used for initial treatment due to the disseminated nature of the disease^18^. Although itraconazole is the drug of choice for immunocompetent individuals due to its superior cure rates and shorter treatment duration compared to sulfamethoxazole-trimethoprim (86.4% vs. 51.3%, 12 vs. 23 months)^19^, there are no direct comparative studies in PLWHA. Sulfamethoxazole-trimethoprim may also be a good option for coinfected individuals, as it has fewer interactions with antiretrovirals^16^ and antituberculous agents. It can also be used as prophylaxis for opportunistic diseases in PLWHA with severe immunosuppression. Establishing good communication with the patient is essential to ensure a clear understanding of both diseases, promote adherence to appropriate treatment regimens, facilitate disease control, and reduce mortality^9^.

## CONCLUSION

Paracoccidioidomycosis, unlike other systemic mycoses, is often not included as a differential diagnosis for opportunistic diseases affecting immunosuppressed patients, particularly those living with HIV/AIDS. The absence of classic risk factors, such as living or working in rural areas, does not exclude the diagnosis, considering that inhalation of conidia from contaminated soil at any stage of life can generate latent foci. Moreover, as with other diseases that share a similar mechanism, it is important to emphasize the need to include PCM as an AIDS-defining illness in endemic areas, given its capacity to reactivate with acute/subacute features in PLWHA. Healthcare professionals should consider early mycological investigations in immunocompromised patients with systemic and cutaneous symptoms to ensure timely diagnosis and management.

## Data Availability

The complete anonymized dataset supporting the findings of this study is included within the article itself.
